# Tubomanometry correlations with patient characteristics and other diagnostic tests of Eustachian tube dysfunction: a cohort study of 432 ears

**DOI:** 10.1007/s00405-022-07358-y

**Published:** 2022-04-06

**Authors:** Heidi Oehlandt, Oskari Lindfors, Saku T. Sinkkonen

**Affiliations:** 1 Department of Otorhinolaryngology–Head and Neck Surgery, Turku University Hospital and University of Turku, Turku, Finland; 2 Department of Otorhinolaryngology–Head and Neck Surgery, Head and Neck Center, Helsinki University Hospital and University of Helsinki, Helsinki, Finland

**Keywords:** Eustachian tube (ET), Eustachian tube dysfunction (ETD), Tubomanometry (TMM), Eustachian tube dysfunction questionnaire-7 (ETDQ-7), Dilatory (obstructive) Eustachian tube dysfunction, Baro-challenge-induced Eustachian tube dysfunction

## Abstract

**Objectives:**

Currently, there is no consensus regarding the best protocol for diagnosing Eustachian tube dysfunction (ETD). We aimed to evaluate how patient characteristics affect tubomanometry (TMM) results. If an association between patient characteristics and TMM results exists, this should be considered in TMM interpretation. We also wanted to study if TMM correlates with other diagnostic tools of ETD.

**Methods:**

A retrospective chart review was conducted on all patients with TMM results available from November 2011 to October 2020 at a tertiary referral center, including 432 ears from 219 patients. An association between diagnostic tests and patient characteristics was assessed using regression models. Spearman’s rank correlation was used to analyze correlations between diagnostic tests.

**Results:**

None of the studied patient characteristics (age, gender, body mass index, smoking, sinonasal disease) was associated with TMM results except for pollen allergy (OR 1.74, 95% CI 1.15–2.63, *P* = 0.009). TMM results correlated with Valsalva maneuver performance (*P* < 0.001, *ρ* = 0.31) and otomicroscopic signs of inadequate middle ear ventilation (*P* < 0.001, *ρ* = 0.28). The Eustachian tube dysfunction questionnaire-7 (ETDQ-7) score did not correlate with any diagnostic method.

**Conclusions:**

TMM results are unaffected by patient characteristics other than pollen allergy. Thus, TMM may be used in ETD diagnostics in a wide variety of patients with straightforward interpretation. TMM correlates with other diagnostic tests studied but not with ETDQ-7. ETDQ-7 does not seem to correlate with other diagnostic tests and appears nonspecific in ETD diagnostics.

## Introduction

The Eustachian tube (ET) is a functional tubular structure connecting the middle ear to the nasopharynx. Its known functions are to equalize the pressure of the middle ear, drain secretions from the middle ear, and protect the middle ear from sounds and pathogens from the nasopharynx. Eustachian tube dysfunction (ETD) can be divided into three subtypes: dilatory (obstructive), baro-challenge-induced (baro-ETD), and patulous ETD [1].

The prevalence of obstructive Eustachian tube dysfunction (OETD) in adults is estimated to be 5% [2]. ETD symptoms such as otalgia, a feeling of pressure, and hearing impairment decrease patients’ quality of life [3]. Chronic OETD can lead to several middle ear pathologies, such as atelectasis of the tympanic membrane (TM), otitis media with effusion, and cholesteatoma [4].

Several tests evaluate ET function, but there is no consensus on the best protocol to diagnose ETD [5]. Otomicroscopy and tympanometry can objectively provide information on middle ear pressure conditions but fail to measure ET function directly. The Valsalva maneuver can give information on ET patency. However, it is non-physiological, and many healthy individuals also fail this test [5, 6]. The Toynbee maneuver is more physiological, but almost half of the healthy individuals fail this test, and the repeatability of the test is inferior to the Valsalva maneuver [6, 7]. ETD questionnaire-7 (ETDQ-7) is a validated scoring system for ETD symptoms but is not an objective measure of ET function [8]. Transnasal video endoscopy allows direct evaluation of the ET’s orifice and can be used to detect pathologies of the orifice and evaluate the muscle activity [9]. However, the entire opening of the ET cannot be evaluated with endoscopy. Sonotubometry assesses sound transmission between nasopharynx and the middle ear through ET and can detect the active opening of ET [10]. Sonotubometry, along with tubomanometry (TMM), is considered the most reliable test for ET patency [11].

In TMM, the principle is to deliver a defined over pressure, typically 30, 40, or 50 mbar, to the nasopharynx. If the ET opens during swallowing, the pressure is transmitted to the middle ear leading to deflection of the tympanic membrane. This changes the pressure in outer ear canal, which can be detected with the TMM device [12].

While the basic principle of TMM is simple and used as an objective and relatively reliable test detecting ET opening [7, 13], it has not gained widespread clinical use. Its use requires education and experience, and clinical interpretation may sometimes be challenging [14]. However, with proper training, the TMM measurement success rate is sufficient for diagnostics [14]. As well as technical difficulties in TMM measurements in inexperienced hands, there seems to be some variability in the results of individual measurements, which cannot always be explained with technical issues. To overcome this variability, TMM results from different pressures may be united as composite scores, such as seven-item Eustachian tube score (ETS-7 score) [15]. ETS-7 includes results from subjective Valsalva and Toynbee maneuvers, objective Valsalva maneuver, tympanometry, and TMM results from 30, 40, and 50 mbars. ETS-7 score ranges from 0 (no tubal function) to 14 (highest tubal function) [15]. Sensitivity and specificity for OETD was 96% in a previous study [15].

Potential contributing factors for OETD have been assumed to be smoking, chronic rhinosinusitis, allergic rhinitis, deviated septum, laryngopharyngeal reflux, and cleft palate. In one prospective study, patients with nasal septum deviation performance worse in TMM and their results improved after septoplasty [16]. An association between abnormal TMM and chronic rhinosinusitis and dust mite allergic rhinitis has been found [17, 18].

To gain confidence in TMM results and their interpretation, revealing possible patient-related contributing factors would be important. This study aimed to evaluate the possible patient characteristics affecting TMM results. To the best of our knowledge, no previous studies on patient characteristics affecting TMM results have been conducted besides those mentioned. We also wanted to study the correlation between TMM and other ETD diagnostic tests to evaluate the reliability of TMM in ETD diagnostics.

## Methods

### Ethics

The Ethics committee of the Hospital District of Helsinki and Uusimaa approved this study (§68/HUS/356/2017).

### Patients and data collection

A retrospective chart review of all consecutive patients visiting our ET outpatient clinic from November 2011 to October 2020 with TMM performed was conducted. Only adult patients (age > 16 yr.) were included. Patients diagnosed with patulous ETD (29 patients, 32 ears), cleft palate (3 patients), or meningioma treated with radiation therapy (1 patient) were excluded. Altogether, 432 ears from 219 patients were included in the study.

Characteristics (gender, age, height, weight, and body-mass-index [BMI, expressed in kg/m^2^]); clinical history (smoking status, allergies, concomitant ear, and sinonasal diseases); ear specific symptoms and clinical findings (TMM results, ETDQ-7 score, otomicroscopic status, and otomicroscopically verified Valsalva maneuvers); and the diagnosis made at the patients’ appointments were retrieved retrospectively from the patients’ charts. The diagnosis was a subjective assessment made by the attending physician based on patient anamnesis, clinical examination, and different diagnostic tests, including TMM. The diagnosis was categorized as normal ET function, baro-ETD, and OETD. Patients were evaluated to have normal ET function if there were no ear symptoms, or the symptoms were not thought to be due to ET dysfunction, as clinical examination or diagnostic tests were not suggestive for ETD.

TMM examinations were conducted using a tubomanometer (Spiggle and Theis, Overath, Germany). TMM measurements were done with predefined nasopharyngeal pressures of 30, 40, and 50 mbar. The ET’s opening was assessed with an *R* value, which describes the ET’s opening latency [13]. *R* < 1 is considered normal, *R* > 1 means delayed ET opening, and no definable R value means no detectable ET opening. In most cases, measurement was started at 30 mbar and increased until normal ET opening was detected using a maximum of 50 mbar. For research purposes, we developed a TMM score to describe the TMM results in a single value (Table 1). In our TMM score, if the patient had ET opening at 30 mbar pressure with normal latency (*R* < 1), the ET function was evaluated as normal, and no further measurements were conducted. In this case, the TMM score for this ear was 0. The ear scored 1 point if the *R* value at 30 mbar was over 1, 2 points when no detectable ET opening was noted at 30 mbar, but at 40 mbar *R* value was < 1, and so forth. The score ranged from 0 to 6 points, ranging from normal ET opening (0 points) to no detectable ET opening at any measured pressures (6 points).

**Table 1:** TMM score used in the current study

TMM score	TMM result at 30 mbar	TMM result at 40 mbar	TMM result at 50 mbar
0	*R* < 1		
1	*R* > 1		
2	No definable *R* value	*R* < 1	
3	No definable *R* value	*R* > 1	
4	No definable *R* value	No definable *R* value	*R* < 1
5	No definable *R* value	No definable *R* value	*R* > 1
6	No definable *R* value	No definable *R* value	No definable *R* value

*TMM* tubomanometry. *R* value describes the opening latency of the eustachian tube (*R* < 1, normal; *R* > 1, delayed; no definable *R* value, no detectable ET opening)

Otomicroscopy had been conducted on all patients. Their status was categorized as normal TM or abnormal TM, with one or more of the following: retraction of pars flaccida, retraction of pars tensa, middle ear effusion, perforation, adhesive otitis media, or a tympanostomy tube. An objective Valsalva maneuver was performed in a sitting or supine position, and in some cases, both, which were otomicroscopically verified by the physician. Valsalva maneuver results were scaled into three categories: positive; weak/delayed; and negative.

### Statistical analysis

Continuous data were tested for skewness. Due to the skewness found, nonparametric tests were used. Statistical differences between patient characteristics in different diagnosis groups were tested using Pearson’s Chi-Square test in categorical data and Kruskal–Wallis test in continuous data (Table 2). Standard deviation (SD) and range are reported in continuous data and the number of ears and percentages in categorical data.

**Table 2:** Patient characteristics, history, and findings

Characteristic	All ears, *N* = *432*	Normal ETF, *N* = *146*	Baro-ETD, *N* = *69*	OETD, *N* = *197*	*P* value
Age (years)	43 ± 15 (16–81)	43 ± 17 (16–81)^a^	38 ± 13 (18–70)	44 ± 15 (16–81)^a^	0.026
Male, Female	179 (41%), 253 (59%)	51 (35%), 95 (65%)	32 (46%), 37 (54%)	85 (43%), 112 (57%)	0.181
BMI (kg/m^2^)	26 ± 5 (16–50)	24 ± 5 (16–37)	25 ± 5 (18–42)	26 ± 5 (18–50)	0.072
Allergy (all)	202 (47%)	58 (40%)	34 (49%)	97 (49%)	0.230
Pollen allergy	118 (27%)	25 (17%)	21 (30%)^a^	65 (33%)^a^	0.005
Smoking	68 (16%)	19 (13%)	1 (1%)	47 (24%)	< 0.001
Concomitant ear disease	101 (23%)	27 (18%)	13 (19%)	56 (28%)	0.062
Concomitant sinonasal disease	78 (18%)	17 (12%)^a^	15 (22%)^a,b^	42 (21%)^b^	0.047
ETDQ-7 score^x^	21 ± 11 (7–48)	19 ± 12 (7–44)^a^	19 ± 10 (7–43)^a^	23 ± 11 (7–48)	0.009
TMM score^w^	2.0 ± 2.2 (0–6)	0.8 ± 1.5 (0–6)	1.4 ± 1.9 (0–6)	3.0 ± 2.3 (0–6)	< 0.001
Otomicroscopy normal	296 (69%)	140 (96%)	58 (84%)	83 (42%)	< 0.001
Retraction of pars tensa	82 (19%)	2 (1%)	6 (9%)	71 (36%)	< 0.001
Retraction of pars flaccida	47 (11%)	3 (2%)^a^	1 (1%)^a^	42 (21%)	< 0.001
Adhesive otitis media	5 (1%)	2 (1%)	0 (0%)	3 (2%)	0.596
Middle ear effusion	33 (8%)	0 (0%)^a^	0 (0%)^a^	33 (17%)	< 0.001
Perforation of tympanic membrane	14 (3%)	1 (1%)^a^	1 (1%)^a,b^	11 (6%)^b^	0.025
Tympanostomy tube	15 (3%)	0 (0%)^a^	0 (0%)^a^	15 (8%)	< 0.001
Objective valsalva: pos./weak/neg	213 (52%)/49 (12%)/147 (36%)	108 (78%)/5 (4%)/26 (19%)	32 (47%) / 7 (10%)/29 (43%)^a^	61 (34%)/35 (19%)/86 (47%)^a^	< 0.001
At supine position: pos./weak/neg	104 (50%)/25 (12%)/77 (37%)	59 (87%)/1 (1%)/8 (12%)	7 (29%)/4 (17%)/13 (54%)^a^	32 (31%)/18 (17%)/54 (52%)^a^	< 0.001
At sitting position: pos./weak/neg	113 (50%)/25 (11%)/90 (39%)	50 (68%)/4 (5%)/19 (26%)^a^	27 (53%)/3 (6%)/21 (41%)^a^	30 (32%)/18 (19%)/46 (49%)	< 0.001

Values were missing for ^x^in 185 ears; ^w^in 66 ears. Categorical data presented as numbers (%); continuous data presented as mean ± SD (range). Categorical data were analyzed using Pearson’s Chi-Square test; continuous were data analyzed using Kruskal–Wallis test. Superscript letters ^a^ and ^b^ denote a subset of diagnosis categories whose column proportions do not significantly differ from each other at the 0.05 level

*Baro-ETD* indicates baro-challenge-induced eustachian tube dysfunction, *BMI* body mass index, *ETDQ-7* eustachian tube dysfunction questionnaire, *N* number of ears, *normal ETF* normal eustachian tube function, *OETD* obstructive eustachian tube dysfunction, *TMM* score tubomanometry score (ranging from 0 to 6). *Objective Valsalva* otomicroscopy-verified Valsalva maneuver at supine, sitting, or at least one of the positions

Association between patient characteristics and diagnostic tools were tested using logistic regression in categorical data and ordinal regression in ordinal scale data. The Valsalva maneuver was categorized as positive (including positive and weak/delayed results) and negative. The diagnosis was categorized as normal or ETD (including baro-ETD and OETD). Valsalva and diagnosis were also tested with multinominal logistic regression to find possible differences in their subgroups. Odds ratio (OR) and 95% confidence interval (95% CI) are reported. Furthermore, if more than one independent variable was statistically significant, multivariable regression was used.

The relationships among diagnostic tests were analyzed using Spearman’s rank correlation, reporting correlation coefficient (*ρ*). Analyses were done with an ordinal scale TMM score. The difference in means was analyzed using the Mann–Whitney *U* test.

In all statistics significance, a *P* value of 0.05 was used. Missing data were excluded pairwise, and the number of included ears (*N*) is reported with *P* values. All statistical calculations were conducted with SPSS Statistics for Windows, version 27 (IBM Corp, Armonk, NY).

## Results

Altogether, 432 ears from 219 patients were included in the study from our ET outpatient clinic. Characteristics of the cohort are shown in Table 2. The cohort’s mean age was 43 years (SD 15.4, ranging from 16 to 81 years). Of the ears, 59% belonged to female patients. At the end of the appointment, 34% (146 ears) were diagnosed as having normal ET function, 16% (69 ears) had baro-ETD, 46% (197 ears) had OETD, and in 5% (20 ears), the diagnosis remained unclear. Of the ears, 69% (*N* = 296) were normal in otomicroscopy, while the rest had one or more signs suggestive of inadequate middle ear ventilation. The otomicroscopy-verified objective Valsalva maneuver was positive in 66% (*N* = 262) of the ears. The mean ETDQ-7 score was 21 (SD 11, ranging from 7 to 48). The mean TMM score was 2 (SD 2.2, ranging from 0 to 6).

### Patient characteristics

The association between patient characteristics and diagnostic test outcomes or ETD diagnosis (including baro-ETD and OETD) are presented in Table 3. Age was not associated with TMM score (*P* = 0.145, *N* = 366). Abnormal TM status was more common in older than younger patients (OR 1.02, 95% CI 1.01–1.03, *P* = 0.006, *N* = 432). Age was not associated between normal ET function and OETD. However, baro-ETD patients were younger than patients with normal ET function (OR 0.98 95% CI 0.96–1.00, *P* = 0.024, *N* = 235). Age and ETDQ-7 or Valsalva maneuver were not associated.

**Table 3:** Correlations between patient characteristics and diagnostic test outcomes or ETD diagnosis

	TMM score (higher score)	ETDQ-7 score (higher score)	TM status abnormal	Valsalva maneuver negative	Diagnosis (baro-ETD or OETD)
Age (OR/year)	OR 1.01 (1.00–1.02)	OR 1.00 (0.99–1.01)	OR 1.02 (1.01–1.03)	OR 1.01 (1.00–1.03)	OR 1.00 (0.98–1.01)
	*P* = 0.145, *N* = 366	*P* = 0.987, *N* = 247	*P = 0.006, *N* = 432	*P* = 0.082, *N* = 409	*P* = 0.529, *N* = 412
Gender (ref. male)	OR 0.74 (0.51–1.07)	OR 1.96 (1.25–3.06)	OR 0.89 (0.59–1.34)	OR 1.97 (1.29–3.02)	OR 0.49 (0.29–0.84)
	*P* = 0.108, *N* = 366	*P = 0.03, *N* = 247	*P* = 0.578, *N* = 432	*P = 0.002, *N* = 409	*P* = 0.074, *N* = 412
BMI (OR/kg/m^2^)	OR 1.02 (0.98–1.05)	OR 0.99 (0.94–1.04)	OR 1.04 (1.00–1.09)	OR 0.99 (0.95–1.03)	OR 1.04 (0.99–1.08)
	*P* = 0.426, *N* = 346	*P* = 0.605, *N* = 233	*P = 0.039, *N* = 412	*P* = 0.492, *N* = 389	*P* = 0.114, *N* = 392
Smoking (ref. no)	OR 1.53 (0.94–2.48)	OR 1.64 (0.88–3.04)	OR 2.53 (1.49–4.28)	OR 0.65 (0.36–1.12)	OR 1.42 (0.80–2.52) ˣ
	*P* = 0.087, *N* = 360	*P* = 0.118, *N* = 239	*P = 0.01, *N* = 424	*P* = 0.148, *N* = 404	*P* = 0.237, *N* = 404
Pollen allergy (ref. no)	OR 1.74 (1.15–2.63)	OR 1.28 (0.80–2.05)	OR 0.75 (0.47–1.20)	OR 1.33 (0.85–2.09)	OR 2.27 (1.37–3.75)
	*P = 0.009, *N* = 362	*P* = 0.310, *N* = 247	*P* = 0.225, *N* = 428	*P* = 0.214, *N* = 405	*P = 0.001, *N* = 408
Animal allergy (ref. no)	OR 1.20 (0.68–2.11)	OR 1.32 (0.70–2.49)	OR 1.14 (0.62–2.10)	OR 1.32 (0.69–2.51)	OR 1.68 (0.86–3.27)
	*P* = 0.522, *N* = 361	*P* = 0.397, *N* = 245	*P* = 0.675, *N* = 426	*P* = 0.396, *N* = 403	*P* = 0.126, *N* = 407
Sinonasal disease (ref. no)	OR 0.83 (0.50–1.37)	OR 1.83 (0.97–3.43)	OR 0.77 (0.44–1.32)	OR 0.79 (0.46–1.35)	OR 2.07 (1.15–3.71)
	*P* = 0.462, *N* = 366	*P* = 0.062, *N* = 247	*P* = 0.339, *N* = 432	*P* = 0.384, *N* = 409	*P = 0.015, *N* = 412

*BMI* body mass index, *diagnosis* Eustachian tube dysfunction diagnosis made by the attending physician (including baro-challenge-induced ETD and obstructive ETD), *ETDQ-7* eustachian tube dysfunction questionnaire, *N* number of patients, *OR* odds ratio (95% CI); *P* *P* value, *TM* tympanic membrane, *TMM* *score* tubomanometry score (ranging from 0 to 6). Statistical analysis was conducted using logistic regression with categorical data (TM, Valsalva, diagnosis) and ordinal regression with ordinal scale data (TMM score, ETDQ-7). ^x^: In multinominal logistic regression, a statistically significant association between smoking and normal ET function and OETD was found (OR 2.01, 95% CI 1.12–3.60, *P* = 0.019)

Gender was not associated with TMM score (*P* = 0.108, *N* = 366). However, female gender was associated with higher ETDQ-7 scores (OR 1.96, 95% CI 1.25–3.06, *P* = 0.030,* N* = 247). Also, the odds of female patients unable to perform the Valsalva maneuver was 1.97 (95% CI 1.29–3.02, *P* = 0.002, *N* = 409) times than with male patients. An association between gender and TM status or diagnosis was not found.

BMI did not associate with TMM score (*P* = 0.426, *N* = 346). An increase in BMI was associated with higher incidence of abnormal TM, with an odds ratio of 1.04 (95% CI 1.00–1.09, *P* = 0.039, *N* = 412). No associations were found between BMI and ETDQ-7, the Valsalva maneuver, or diagnosis groups.

Smoking was not associated with TMM score (*P* = 0.087, *N* = 360). However, current smokers had higher odds of OETD diagnosis than normal ET function (OR 2.01, 95% CI 1.12–3.60, *P* = 0.019, *N* = 336). Smoking was also associated with higher incidence of abnormal TM status (OR 2.53, 95% CI 1.49–4.28, *P* = 0.01, *N* = 424), more specifically with retraction of the pars flaccida (OR 2.87, 95% CI 1.45–5.65, *P* = 0.002) and retraction of the pars tensa (OR 3.05, 95% CI 1.73–5.39, *P* < 0.001). Smoking was not associated with ETDQ-7 or the Valsalva maneuver.

Pollen allergy was associated with higher TMM scores (OR 1.74, 95% CI 1.15–2.63, *P* = 0.009, *N* = 362). The median TMM score in patients with a pollen allergy was 2 (IQR = 5), compared to 1 (IQR = 3), in non-allergic patients. Also, the odds of an ETD diagnosis were higher if patient had a pollen allergy (OR 2.27, 95% CI 1.37–3.75, *P* = 0.001, *N* = 408). The same association was not found with animal allergy. Also, no associations were found between pollen or animal allergy and ETDQ-7, the Valsalva maneuver, or TM status.

Sinonasal disease was not associated with TMM score (*P* = 0.462, *N* = 366). However, sinonasal disease was associated with ETD diagnosis (OR 2.07, 95% CI 1.15–3.71, *P* = 0.015, *N* = 412). Significant association was found between normal ET and OETD (OR 2.06, 95% CI 1.12–3.78, *P* = 0.021, *N* = 343) but not between normal ET and baro-ETD. Sinonasal disease was not associated with ETDQ-7, the Valsalva maneuver, or TM status.

Multivariable logistic regression was performed to ascertain the effect of age, BMI, and smoking on the likelihood of patients having an abnormal tympanic membrane. The model explained 9.7% (Nagelkerke *R*^2^) of the variance in TM status (*χ*^2^(3) = 29.29, *P* < 0.001). A test was also performed to ascertain the effect of smoking, pollen allergy, and sinonasal disease on the likelihood of OETD diagnosis compared to normal ET function. The model explained 9.0% (Nagelkerke *R*^2^) of the variance in diagnosis (*χ*^2^(3) = 22.85, *P* < 0.001).

### Correlations between different diagnostic tests

**Fig. 1:**
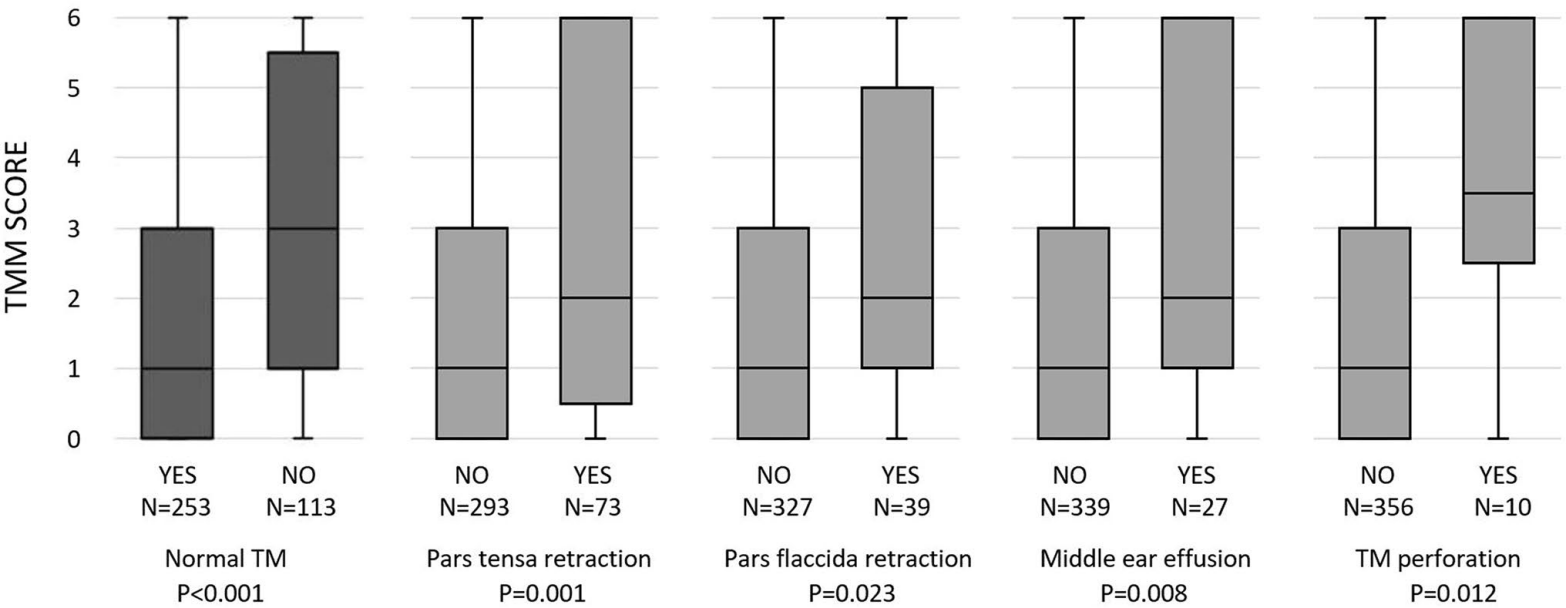
Box plot of the association between patient tympanic membrane status and TMM score. *TMM score* tubomanometry result (values from 0 = normal ET opening to 6 = no ET opening at 30–50 mbar pressure). *N* number of ears, *P P* value, *TM* tympanic membrane. Statistical analysis was conducted using the Mann–Whitney *U* test

Correlations between different diagnostic tests are shown in Table 4. Higher TMM score correlated to abnormal TM findings (*ρ* = 0.283, *P* < 0.001, *N* = 366). Median TMM score was 1 (IQR = 3) if TM was normal and 3 (IQR = 5) if TM was abnormal. Association was found with pars tensa retraction, pars flaccida retraction, middle ear effusion, and perforation of the tympanic membrane (all *P* values < 0.05). More detailed information about the associations is presented in Fig. 1. TMM results also correlated with objective Valsalva maneuver performance (*ρ* = 0.306, *P* < 0.001, *N* = 349). Valsalva maneuver performance in supine position appears to be more sensitive (supine: *ρ* = 0.445, *P* < 0.001, *N* = 177 and sitting: *ρ* = 0.229, *P* = 0.001, *N* = 195). A correlation between TMM score and ETDQ-7 score was not found (*ρ* = 0.086, *P* = 0.217, *N* = 207). An association to the TMM score was not found even if the results of ETDQ-7 score were classified as normal (score ≤ 14) or abnormal (score > 14) (*P* = 0.342, *N* = 207).

**Table 4:** Correlations between different diagnostic tests

Test	*P* value	*N*	Effect size
TMM score AND			
TM status	< 0.001	366	*ρ* = 0.283
Valsalva maneuver	< 0.001	349	*ρ* = 0.306
ETDQ-7 score	0.217	207	*ρ* = 0.086
ETDQ-7 score AND			
TM status	0.185	247	*ρ* = 0.085
Valsalva maneuver	0.073	234	*ρ* = 0.117
Valsalva maneuver AND			
TM status	< 0.001	409	*ρ* = 0.279

*ETDQ-7* eustachian tube dysfunction questionnaire, *N* number of ears, *TM* tympanic membrane, *TMM* *score* tubomanometry score (ranging from 0 to 6), *ρ* correlation coefficient. Statistical analysis conducted with Spearman’s rank correlation

ETDQ-7 score did not correlate with TM status or objective Valsalva maneuver (*ρ* = 0.085, *P* = 0.185, *N* = 247, and *ρ* = 0.117, *P* = 0.073, *N* = 234, respectively). Objective Valsalva maneuver correlated with abnormal TM status (*ρ* = 0.279, *P* < 0.001, *N* = 409). In 60%, the TM was normal if objective Valsalva maneuver was positive, 12% if Valsalva was weak, and 28% if Valsalva was negative (*P* < 0.001, *N* = 409).

## Discussion

Patient characteristics (age, gender, BMI, smoking, or sinonasal disease) other than pollen allergy did not affect TMM score in the current study. This suggests that TMM results may be interpreted as independent of patient characteristics, making clinical use of TMM much easier. TMM score correlated with otomicroscopic findings and Valsalva maneuver performance. In a previous study, using TMM in clinical diagnostic had an overall success rate of 91% [14]. Based on these results, we believe TMM is a reliable and useful diagnostic tool for all ETD patients.

Patients with a pollen allergy had higher TMM scores. An association between pollen allergy and OETD has been shown in studies [19, 20]. Another assumed risk factor for OETD is smoking. One small study found a link between smoking and poor ET function [21], and another found a link between smoked or non-smoked tobacco and middle ear diseases in adult patients [22]. In children, evidence of tobacco smoke exposure as a risk factor for middle ear diseases and possible OETD is stronger [23, 24]. However, in our study, tests of ET patency (TMM and Valsalva maneuver) showed no association between smoking and ET function, though TM retraction was more common in smokers. In our study, TM findings were more common in older patients and patients with higher BMI. However, an association between those characteristics and outcome of the ET function tests was not found (TMM, Valsalva maneuver, and ETDQ-7 score).

Based on this patient cohort, ETDQ-7 is not a valid diagnostic tool for ETD because it did not correlate with other ET tests (TMM, otomicroscopy, or Valsalva maneuver). The same conclusion was reached in a recent review article [8].

### Limitations

Due to this study’s retrospective nature, some patient data were missing. In some cases, if ET opened normally at 30 mbar, no further measurements were done. Hence, a TMM score was formed to assess ET function on an ordinal scale. A similar score system has not been used in previous studies. At early stages of the study period, ETDQ-7 was not done separately for both ears. In this cohort, we analyzed ears individually, so we dismissed those results. After April 2018, ETDQ-7 was done for both ears separately in our ET outpatient clinic.

## Conclusion

TMM is unaffected by patient characteristics other than a pollen allergy. TMM results correlate with otomicroscopy findings and Valsalva maneuver performance but not with the ETDQ-7 score. Thus, TMM interpretation in ETD is straightforward irrespective of the patient characteristics.

## References

[1] Schilder AGM, Bhutta MF, Butler CC (2015). Eustachian tube dysfunction: consensus statement on definition, types, clinical presentation and diagnosis. Clin Otolaryngol.

[2] Shan A, Ward BK, Goman AM (2019). Prevalence of eustachian tube dysfunction in adults in the United States. JAMA Otolaryngol—Head Neck Surgery.

[3] Teklu M, Kulich M, Micco AG (2020). Measuring the health utility of chronic eustachian tube dysfunction. Laryngoscope.

[4] Seibert JW, Danner CJ (2006). Eustachian tube function and the middle ear. Otolaryngol Clin North Am.

[5] Smith ME, Tysome J (2015). Tests of eustachian tube function: a review. Clin Otolaryngol.

[6] Hidir Y, Ulus S, Karahatay S, Satar B (2011). A comparative study on efficiency of middle ear pressure equalization techniques in healthy volunteers. Auris Nasus Larynx.

[7] Smith ME, Zou CC, Baker C (2017). The repeatability of tests of eustachian tube function in healthy ears. Laryngoscope.

[8] Andresen NS, Sharon JD, Nieman CL (2021). Predictive value of the eustachian tube dysfunction questionnaire-7 for identifying obstructive eustachian tube dysfunction: a systematic review. Laryngoscope Investig Otolaryngol.

[9] Poe DS, Pyykkö I (2011). Measurements of eustachian tube dilation by video endoscopy. Otol Neurotol.

[10] van der Avoort SJC, van Heerbeek N, Zielhuis GA, Cremers CWRJ (2005). Sonotubometry: eustachian tube ventilatory function test: a state-of-the-art review. Otol Neurotol.

[11] Smith ME, Takwoingi Y, Deeks J (2018). Eustachian tube dysfunction: a diagnostic accuracy study and proposed diagnostic pathway. PLoS ONE.

[12] Esteve D (2003). Tubomanometry and pathology fibrocartilaginous eustachian tube: middle ear cleft.

[13] Schröder S, Lehmann M, Korbmacher D (2015). Evaluation of tubomanometry as a routine diagnostic tool for chronic obstructive eustachian tube dysfunction. Clin Otolaryngol.

[14] Lindfors OH, Oehlandt H, Sinkkonen ST (2021). Tubomanometry measurement success rate in clinical practice. Otol Neurotol.

[15] Schröder S, Lehmann M, Sauzet O (2015). A novel diagnostic tool for chronic obstructive eustachian tube dysfunction-the eustachian tube score. Laryngoscope.

[16] Fontes Lima A, Carvalho Moreira F, Esteves Costa I (2021). Nasal septum deviation and eustachian tube function: a prospective case-control study based on tympanometry, tubomanometry, and ETDQ-7. Acta Otorrinolaringologica Espanola.

[17] Ma Y, Liang M, Tian P (2020). Eustachian tube dysfunction in patients with house dust mite-allergic rhinitis. Clin Transl Allergy.

[18] Vandersteen C, Castillo L, Roger C (2021). Tubomanometry: an effective and promising assessment of eustachian tube dysfunction in chronic rhinosinusitis patients. Eur Ann Otorhinolaryngol Head Neck Dis.

[19] Lazo-Sáenz JG, Galván-Aguilera AA, Martínez-Ordaz VA (2005). Eustachian tube dysfunction in allergic rhinitis. Otolaryngol Head Neck Surg.

[20] Juszczak H, Aubin-Pouliot A, Sharon JD, Loftus PA (2019). Sinonasal risk factors for eustachian tube dysfunction: cross-sectional findings from NHANES 2011–2012. Int Forum Allergy Rhinol.

[21] Pezzoli M, Lofaro D, Oliva A (2017). Effects of smoking on eustachian tube and hearing. Int Tinnitus J.

[22] Gaur K, Kasliwal N, Gupta R (2012). Association of smoking or tobacco use with ear diseases among men: a retrospective study. Tob Induc Dis.

[23] Patel MA, Mener DJ, Garcia-Esquinas E (2016). Tobacco smoke exposure and eustachian tube disorders in US children and adolescents. PLoS ONE.

[24] Jones LL, Hassanien A, Cook DG (2012). Parental smoking and the risk of middle ear disease in children: a systematic review and meta-analysis. Arch Pediatr Adolesc Med.

